# Can Positron Emission Tomography/Computed Tomography with the Dual Tracers Fluorine-18 Fluoroestradiol and Fluorodeoxyglucose Predict Neoadjuvant Chemotherapy Response of Breast Cancer? ----A Pilot Study

**DOI:** 10.1371/journal.pone.0078192

**Published:** 2013-10-21

**Authors:** Zhongyi Yang, Yifei Sun, Jing Xue, Zhifeng Yao, Junyan Xu, Jingyi Cheng, Wei Shi, Beiling Zhu, Yongping Zhang, Yingjian Zhang

**Affiliations:** Department of Nuclear Medicine, Fudan University Shanghai Cancer Center, Department of Oncology, Shanghai Medical College, Fudan University, Shanghai, China; University Medical Centre Utrecht, The Netherlands

## Abstract

**Objective:**

To assess the clinical value of dual tracers Positron emission tomography/computed tomography (PET/CT) ^18^F-fluoroestradiol (^18^F-FES) and ^18^F-fluorodeoxyglucose (^18^F-FDG) in predicting neoadjuvant chemotherapy response (NAC) of breast cancer.

**Methods:**

Eighteen consecutive patients with newly diagnosed, non-inflammatory, stage II and III breast cancer undergoing NAC were included. Before chemotherapy, they underwent both ^18^F-FES and ^18^F-FDG PET/CT scans. Surgery was performed after three to six cycles of chemotherapy. Tumor response was graded and divided into two groups: the responders and non-responders. We used the maximum standardized uptake value (SUVmax) to qualify each primary lesion.

**Results:**

Pathologic analysis revealed 10 patients were responders while the other 8 patients were non-responders. There was no statistical difference of SUVmax-FDG and tumor size between these two groups (*P*>0.05). On the contrary, SUVmax-FES was lower in responders (1.75±0.66 versus 4.42±1.14; U=5, *P*=0.002); and SUVmax-FES/FDG also showed great value in predicting outcome (0.16±0.06 versus 0.54±0.22; U=5, *P*=0.002).

**Conclusions:**

Our study showed ^18^F-FES PET/CT might be feasible to predict response of NAC. However, whether the use of dual tracers ^18^F-FES and ^18^F-FDG has complementary value should be further studied.

## Introduction

Neoadjuvant Chemotherapy (NAC) has been one of the standard therapies for the treatment of stage II and III breast cancer [1-3]. However, about 60%-90% of patients achieve clinical response, and complete pathological response is noted in 3%-30% of patients in most breast cancer trials [4,5]. Thus, one of the greatest needs in NAC of breast cancer is to find an early and accurate way to determine which patients are responding to therapy to avoid the toxicity and cost of ineffective therapy and to allow a change to more effective treatment for the individual patient. This is central to the concept of personalized medicine [6]. 

Previous studies demonstrated that pathological features including grade, the degree of expression of estrogen (ER), progesterone receptors (PR) and markers of cell proliferation such as Ki67 labeling index, could be applied to identify tumor subtypes associated with different responses to NAC [7]. However, these features, such as ER status, may be discordant within the same patient. Aitken et al once reported a high discordant rate between primary and nodal disease. Hence, a single biopsy may not be adequate for making a treatment decision [8]. 

Positron emission tomography (PET) allows noninvasive visualization and quantitative assessment of many biologic processes. Furthermore, whether multiple new PET tracers could also predict early response to therapy is being evaluated [6]. Hereby, the purpose of our pilot study was to assess the clinical value of dual tracers PET/CT ^18^F-FES and ^18^F-FDG in predicting NAC response of breast cancer.

## Materials and Methods

### Patients

Fudan University Shanghai Cancer Center ethics committee approved the prospective study and all patients gave their written informed consent before enrollment. From February to December 2012, eighteen consecutive patients with newly diagnosed, non-inflammatory, stage II and III invasive ductal breast carcinomas scheduling to undergo NAC were included. Diagnosis of invasive breast carcinoma was done by core needle biopsy (CNB) in all patients and the stage of cancer was determined by x-ray, abdominal ultrasonography and bone scan investigations according to TNM classification (version 7) [9]. Exclusion criteria were the following: pregnancy, prior breast surgery, chemotherapy, or radiation therapy; known diabetes; age younger than 18 years; unwillingness or inability to undergo both ^18^F-FES and FDG PET/CT scans before NAC; or ineligibility for surgery.

### NAC and surgery

All patients underwent three to six cycles of NAC with paclitaxel (80 mg/m^2^) and carboplatin (AUC 2mg*min/ml) on day 1, 8, and 15 of a 28-day cycle. No other anti-cancer treatments, including chemotherapy, radiation therapy or endocrine therapy before surgery, were permitted. After completion of the NAC, all patients underwent mastectomy with axillary lymph node dissection. The decision of the details of the treatment was made by the multidisciplinary of breast cancer in our hospital. 

### PET/CT imaging

Patients underwent both ^18^F-FES and ^18^F-FDG PET/CT before NAC. The interval between these two scans was less than 7 days.

^18^F-FES was prepared according to published methods [10], and modified by us, as reported in our prior study [11]. ^18^F-FDG was produced automatically by cyclotron (Siemens CTI RDS Eclips ST, Knoxville, Tennessee, USA) using Explora FDG_4_ module in our center. 

^18^F-FES and ^18^F-FDG PET/CT studies were performed on 2 separate days. For the FES study, a typical injection involved 6mCi (222MBq) of radiopharmaceutical in 20ml of isotonic phosphate buffered saline with less than 7% of ethanol content (average dose: 208MBq, range 178-222MBq), and it was administered intravenously over 1min. Scanning was initiated 1h after administration of the tracer. The images were obtained on a Siemens biograph 16HR PET/CT scanner (Knoxville, Tennessee, USA). The transaxial intrinsic spatial resolution was 4.1 mm (full-width at half-maximum) in the center of the field of view. The data acquisition procedure was as follows: CT scanning was first performed, from the proximal thighs to head, with 120kV, 80-250mA, pitch 3.6, rotation time 0.5. Immediately after CT scanning, a PET emission scan that covered the identical transverse field of view was obtained. Acquisition time was 2-3 min per table position. PET image data sets were reconstructed iteratively by applying the CT data for attenuation correction, and coregistered images were displayed on a workstation.

Before the ^18^F-FDG PET/CT, all the patients were requested to fast at least 4h. At the time of the tracer injection (dosage: 7.4MBq/kg), the patients presented blood glucose level under 10mmol/L. Before and after injection, patients were kept lying comfortably in a quiet, dimly lit room. The parameters of PET/CT were the same as ^18^F-FES PET/CT scans.

### Image Interpretation

A multimodality computer platform (Syngo, Siemens, Knoxville, Tennessee, USA) was used for image review and manipulation. Two experienced nuclear medicine physician evaluated the images independently and were unaware of the details regarding clinical and pathologic tumor responses. The reviewers reached a consensus in cases of discrepancy.

Quantification of tumor metabolic activity was obtained using the maximum Standardized Uptake Value (SUVmax) normalized to body weight. The uptake in each primary breast lesion was calculated for further analysis.

### Pathological assessment and Immunohistochemistry

All pathological assessment and immunohistochemistry were performed at our hospital. Immunohistochemistry (IHC) analysis was performed on formalin-fixed, paraffin-embedded tissue sections using standard procedures for breast tumor specimens from CNB to evaluate the expression of ER, PR, human epidermal growth factor receptor-2 (HER-2) and Ki67 prior to NAC. The cut-off value for ER positivity and PR positivity was 1% positive tumor cells with nuclear staining. HER-2 was evaluated as 0, 1+, 2+ or 3+ using circumferential membrane-bound staining. The Ki-67 value was expressed as the percentage of positive cells (at least 1,000) with nuclear staining in each case. The following antibodies were used for IHC: ER (M7047, clone 1D5, Dako, Produktionsvej, Glostrup, Denmark), PR (M3569, clone PR 636, Dako), HER-2 (A0485, polyclonal rabbit antibody, Dako) and Ki-67 (M7240, clone MIB-1, Dako).

To assess pathologic response, fresh surgical specimens were cut in slices of approximately 0.5cm in thickness and examined for the presence or absence of macroscopic tumors. Complete tumors or tumor sites were sampled. When no macroscopic lesion was visible, a large number of sections were analyzed. Specimens were fixed in 10% formaldehyde and embedded in paraffin. Five-micrometer-thick sections were stained with hematoxylin, eosin, and saffron. Tumor response was assessed by a pathologist and graded according to the scale established by Sataloff: total or near-total therapeutic effect (grade A), more than 50% therapeutic effect but less than total or near-total effect (grade B), less than 50% therapeutic effect but visible effect (grade C), or no therapeutic effect (grade D). Therapeutic effect is defined by microscopic changes as fibrous stroma, necrosis, calcifications, or foamy macrophages with or without inflammatory infiltration [12]. Pathologic tumor regression was used as the gold standard to evaluate treatment response. Two major regression groups, the responders (grades A+B) and non-responders (grades C+D), were thus defined according to previous study [13]. 

### Statistical Analysis

The ^18^F-FES and FDG uptake was compared with pathological features (tumor size, ER, PR, HER2 and Ki67%) and the results were reported by use of Spearman correlation coefficients. Comparisons between responder and non-responder groups were made using non-parametric tests (Mann Whitney U test). Data was analyzed by SPSS 13.0 software. All analyses were two-sided. A *P* value less than 0.05 was taken to indicate a significant difference.

## Results

### Patient characteristics and pathological response

The characteristics of the 18 enrolled breast cancer patients were listed in table 1. All the IHC assessments were obtained from the biopsy before NAC. The median primary tumor size was 4.5cm (ranging from 2 to 12cm). According to TNM staging, eight patients were stage II and the other ten were stage III. After completion of chemotherapy, based on pathological findings, 10 patients were considered to be responders (grade A=4; grade B=6) while the rests were non-responders (grade C=8). 

**Table 1:** The characteristics, PET/CT and pathological results of these 18 enrolled breast cancer patients.

No	Age	Cycle	ER	PR	HER2	Ki67%	Stage	Maximum Diameter (cm)	SUVmax-FES	SUVmax-FDG	Ratio	Diagnosis
1	43	4	0	0	2	60	IIB	3.2	1.1	8.99	0.12	R
2	57	6	0	0	3	10	IIB	3.5	1.2	8.6	0.14	R
3	53	4	0	0	3	40	IIIA	6.9	1.3	7.2	0.18	R
4	28	4	2	1	2	50	IIIA	4.3	1.86	27.43	0.07	R
5	52	3	1	0	1	40	IIIA	5.1	2.06	13.88	0.15	R
6	62	4	0	0	3	30	IIB	4.6	1.4	6.06	0.23	R
7	49	4	3	3	2	40	IIIA	4.3	3.39	12.24	0.28	R
8	64	3	0	0	3	50	IIIA	7.5	1.01	15.8	0.06	R
9	49	3	3	3	2	50	IIIA	5.0	2.98	11.08	0.27	R
10	38	3	0	0	0	70	IIA	2.0	1.18	9.51	0.12	R
11	57	6	0	0	3	30	IIIB	12	2.35	16.63	0.14	NR
12	56	3	3	2	2	60	IIIA	4.8	5.1	11.96	0.43	NR
13	65	3	3	0	1	20	IIB	4.9	4.54	8.23	0.55	NR
14	65	4	2	2	2	50	IIIA	5.3	4.3	13.51	0.32	NR
15	48	3	3	2	2	20	IIA	2.7	2.05	5.15	0.4	NR
16	44	3	3	3	2	40	IIIA	3.8	5.91	7.3	0.81	NR
17	63	4	3	2	0	60	IIA	2.2	5.3	6.8	0.78	NR
18	64	4	3	3	1	20	IIA	2.1	5.8	6.3	0.92	NR

R=responder; NR=non-responder

### The correlations of ^18^F-FES, ^18^F-FDG uptake and pathological features

There was good agreement between ^18^F-FES uptake and ER status (coefficient=0.819, *P*<0.001). In addition, ^18^F-FES was also associated with PR (coefficient=0.736, *P*<0.001). As for the other features (HER2, Ki67% and tumor size), there were no correlations (*P*>0.05). Besides, there was no statistical agreement between ^18^F-FDG uptake and these pathological features (*P*>0.05). 

### Treatment response predicted by PET/CT

**Figure 1:**
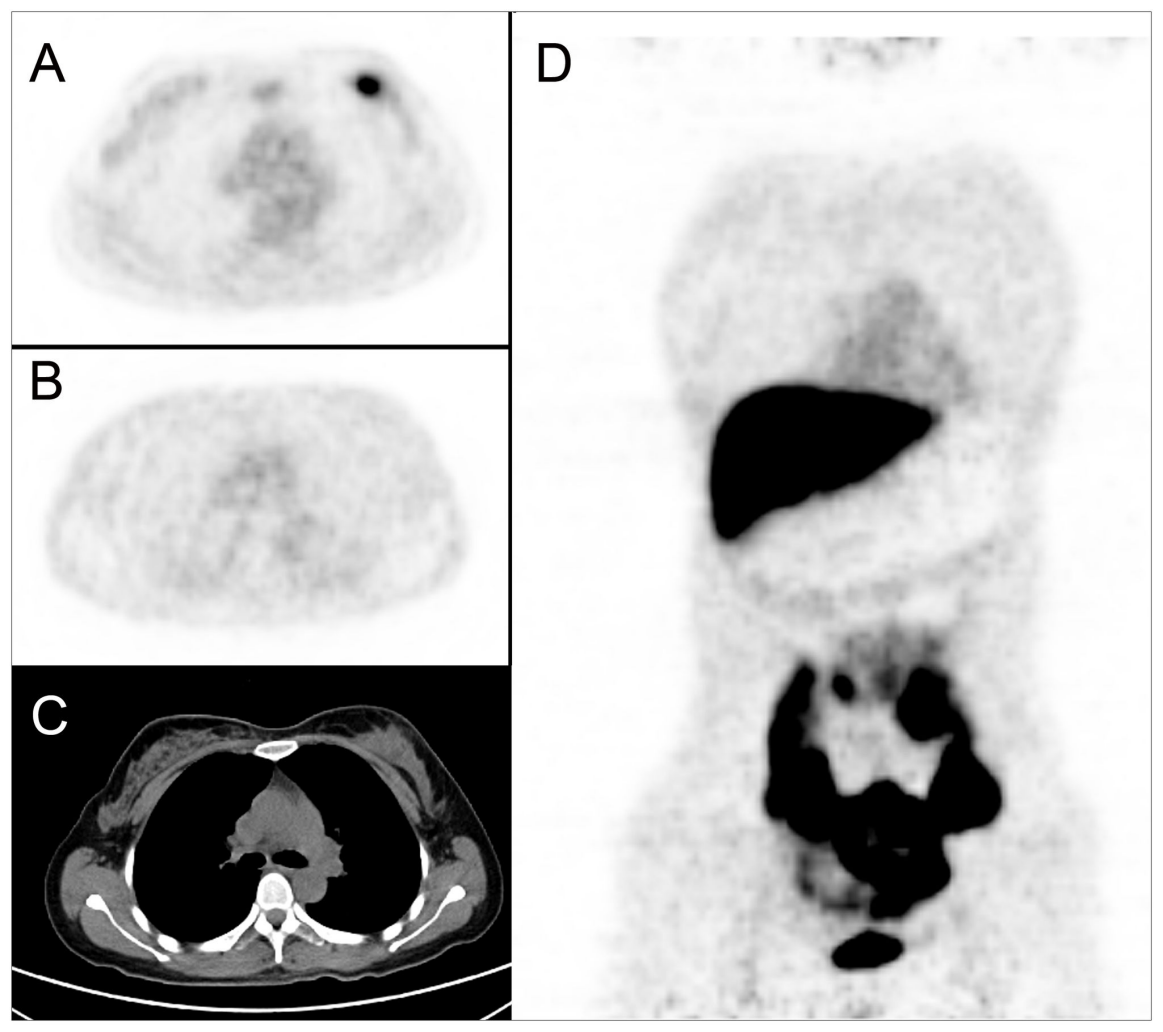
A 43-year-old female breast cancer patient scheduled to undergo adjuvant chemotherapy. We detected a mass in left breast (C. CT imaging), with a maximum diameter of 3.2cm. The tumor was FDG-avid with SUVmax of 8.99 (A. FDG PET/CT), but no obvious FES uptake was noted (B, D. FES PET/CT, SUVmax=1.1). After 4 cycles of NAC, the patient had mastectomy and the pathology showed it was grade A.

**Figure 2:**
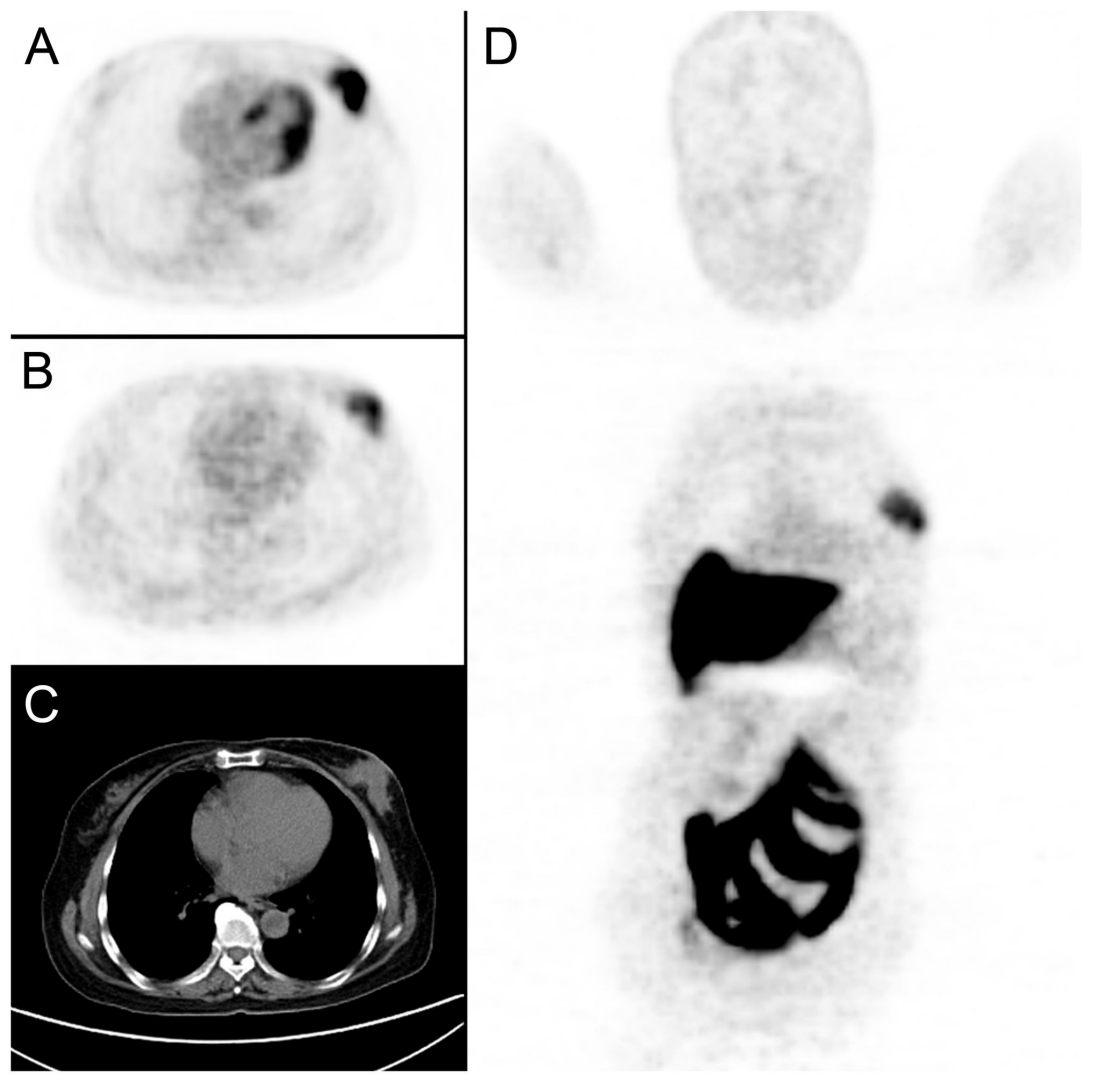
A 65-year-old female breast cancer patient underwent both ^18^F-FDG and FES PET/CT before NAC. A tumor was detected in left breast (C. CT imaging, diameter=5.3cm), with high FDG (A. FDG imaging, SUVmax=13.51) and FES uptake (B, D. FES imaging, SUVmax=4.3). After surgery, the pathological result was confirmed to be grade C.

We found that SUVmax-FES was lower in responders (1.75±0.66 versus 4.42±1.14; U=5, *P*=0.002); and SUVmax-FES/SUVmax-FDG also showed great value in predicting prognosis (0.16±0.06 versus 0.54±0.22; U=5, *P*=0.002). If we used an arbitrary cut-off value of 0.3 (ratio of SUVmax-FES/FDG), the sensitivity and specificity of predicting NAC response were 100% and 87.5%, respectively (Figure 1 and 2). However, SUVmax-FDG did not show difference between these two groups (*P*>0.05). 

## Discussion

It has been shown that after completion of chemotherapy, patients with minimal residual disease confirmed by pathology have a significantly improved disease-free survival and overall survival than those with gross residual disease [5]. Therefore, accurate assessment of the response to NAC is one of the critical factors in optimizing a chemotherapy regimen and planning further surgery. However, conventional imaging modalities, such as mammography and ultrasound were limited in predicting a pathological complete response [14]. 

As a molecular imaging, residual tumor FDG uptake after completing NAC could predict residual disease and is highly predictive of relapse [15-19]. Moreover, early prediction of tumor response during the course of NAC using serial FDG PET scans has been most widely evaluated during the past 10 years, using a comparison to histopathology assessment of response from the post-surgery specimen as a gold standard. In particular, the change in tumor glucose metabolism between baseline and after one or two cycles of NAC is significantly correlated with histopathologic response after the completion of therapy [6,13]. In addition, magnetic resonance imaging (MRI) also provides functional imaging techniques like dynamic contrast-enhanced magnetic resonance imaging [20], diffusion-weighted imaging [21], and magnetic resonance spectroscopy [22], which have all shown promising results as surrogate markers of response to NAC of breast cancer. However, whether ^18^F-FDG PET or MRI is more appropriate to reflect the response of NAC is still controversial. Furthermore, these methods predicted the response at least one or two cycles of NAC, which would cause the potential toxicity and higher cost. Hence, we would like to find a new way to more early predict the prognosis of NAC.

Tan suggested that patients with ER-negative, PR-negative and HER2-amplified breast cancer phenotypes were more likely to experience pathological complete response to NAC [23]. Prechts’ study had the similar consequence [24]. Recently, Houssami et al performed a meta-analysis of 30 studies and also proved that the patients with triple-negative tumors would most benefit from NAC [25]. Hence, detecting these features is crucial for treatment decision. ER expression is routinely measured in clinical practice by in vitro assay of biopsy material, but there exists several limitations: The technique is semi-quantitative, and a standardized molecular class is lacking, which will lead to inter-observer variation; Besides, ER scoring strongly depends on the antibody used and delay-to-fixation time [26,27]. The American Society of Clinical Oncology and the College of American Pathologists reported striking data that up to 20% of all IHC determinations worldwide may be inaccurate. Most of the issues with testing have occurred due to variation in pre-analytic variables, thresholds for positivity, and interpretation criteria [28]. Additionally, ER expression can be heterogeneous sometimes even in one patient with multiple lesions; in addition, receptor expression may change after treatment [8,29]. 

PET with ER-targeting radiopharmaceuticals is a noninvasive method for assessing regional ER-expression in vivo. Several studies have shown that ^18^F-FES PET can reliably detect ER-positive tumor lesions and that ^18^F-FES uptake correlates well with IHC scoring for ER [30]. Our study confirmed these findings. ^18^F-FES PET has been widely used in breast cancer patients. It has been shown that initial ^18^F-FES uptake measurements could be used to predict outcome of patients with ER-positive tumors who underwent hormonal therapy [31,32]. Moreover, it could be a valuable additional diagnostic tool when standard work-up is inconclusive: such as in detection of metastases or guiding treatment decisions [33]. 

As a molecular-imaging modality, PET, by using SUVmax, may provide a more objective representation of the primary lesion, which perhaps may lead to early prediction of the prognosis after NAC. Although ^18^F-FES alone could predict the outcome in our pilot study, we considered that adding FDG information would be more appropriate. The reasons are listed below: In Kurland and colleagues’ study, it was demonstrated that low ratio of ^18^F-FES and FDG SUVmax suggested the highly proliferative, ER poorly function disease that may be better suited to cytotoxic chemotherapy; and additionally, it may also control for partial-volume effects and for effects of different adjustments for distribution volume [34]. Consequently, we used this ratio in order to predict the NAC response. Inspiringly, we found even in the limited population, there was statistical difference of the ratio between responders and non-responders. Therefore, we presumed that tumor with high ratio ^18^F-FES and FDG SUVmax may benefit more from neoadjuvant endocrine therapy than NAC.

However, there are several limitations that are worth mentioning. The first is the limited number of patients. Since it was a pilot study, we only enrolled eighteen patients, which cannot demonstrate our result sufficiently; Secondly, all of our patients were Chinese; the consequence may be different when compared with other races, thus limiting the generalizability of the results. Thirdly, all enrolled patients were treated using a paclitaxel/carboplatin regimen; therefore, it is possible that the results may only apply to the population described in our study.

## Conclusions

Although our pilot study with limited number of patients showed that the use of ^18^F-FES PET alone may be feasible to differentiate responders from non-responders to neoadjuvant chemotherapy, it cannot yet be excluded that the use of dual tracers ^18^F-FES and ^18^F-FDG has complementary value. Clinical trials, especially prospective and multi-center studies with larger number of patients should be performed to validate these findings. 
